# Entomological indicators of malaria transmission prior to a cluster-randomized controlled trial of a ‘lethal house lure’ intervention in central Côte d’Ivoire

**DOI:** 10.1186/s12936-022-04196-5

**Published:** 2022-06-15

**Authors:** Rosine Z. Wolie, Alphonsine A. Koffi, Leslie Ayuk-Taylor, Ludovic P. Ahoua Alou, Eleanore D. Sternberg, Oulo N’Nan-Alla, Yao N’Guessan, Amal Dahounto, Welbeck A. Oumbouke, Innocent Z. Tia, Simon-Pierre A. N’Guetta, Jackie Cook, Matthew B. Thomas, Raphael N’Guessan

**Affiliations:** 1 Unité de Recherche Et de Pédagogie de Génétique, Université Félix Houphouët-Boigny, UFR Biosciences, Abidjan, Côte d’Ivoire; 2 Vector Control Product Evaluation Centre-Institut Pierre Richet (VCPEC-IPR), Institut Pierre Richet (IPR), Bouaké, Côte d’Ivoire; 3 Institut Pierre Richet (IPR), Institut National de Santé Publique (INSP), Bouaké, Côte d’Ivoire; 4 DC Department of Health, 899 North Capitol St NE, Washington, USA; 5 Department of Entomology, Center for Infectious Disease Dynamics, The Pennsylvania State University, University Park, PA USA; 6 Department of Vector Biology, Liverpool School of Tropical Medicine, Liverpool, L3 5QA UK; 7 Department of Disease Control, London School of Hygiene and Tropical Medicine, London, UK; 8 Université Alassane Ouattara, Bouaké, Côte d’Ivoire; 9 Innovative Vector Control Consortium, IVCC, Liverpool, UK; 10 Department of Infectious Disease Epidemiology, International Statistics and Epidemiology Group, London School of Hygiene and Tropical Medicine, London, UK; 11 York Environmental Sustainability Institute, University of York, York, UK

**Keywords:** Malaria transmission, *Anopheles*, *Plasmodium*, Insecticide resistance genes, Côte d’Ivoire

## Abstract

**Background:**

A study was conducted prior to implementing a cluster-randomized controlled trial (CRT) of a lethal house lure strategy in central Côte d’Ivoire to provide baseline information on malaria indicators in 40 villages across five health districts.

**Methods:**

Human landing catches (HLC) were performed between November and December 2016, capturing mosquitoes indoors and outdoors between 18.00 and 08.00 h. Mosquitoes were processed for entomological indicators of malaria transmission (human biting, parity, sporozoite, and entomological inoculation rates (EIR)). Species composition and allelic frequencies of *kdr-w* and *ace-1*^*R*^ mutations were also investigated within the *Anopheles gambiae* complex.

**Results:**

Overall, 15,632 mosquitoes were captured. *Anopheles gambiae *sensu lato (*s.l*.) and *Anopheles funestus* were the two malaria vectors found during the survey period, with predominance for *An. gambiae* (66.2%) compared to *An. funestus* (10.3%). The mean biting rate for *An. gambiae* was almost five times higher than that for *An. funestus* (19.8 bites per person per night for *An. gambiae vs* 4.3 bites per person per night for *An. funestus*) and this was evident indoors and outdoors. *Anopheles funestus* was more competent to transmit malaria parasites in the study area, despite relatively lower number tested for sporozoite index (4.14% (63/1521) for *An. gambiae * vs 8.01% (59/736) for *An. funestus*; χ^2^ = 12.216; P < 0.0001). There were no significant differences between the proportions infected outdoors and indoors for *An. gambiae* (4.03 *vs* 4.13%; χ^2^ = 0.011; P = 0.9197) and for *An. funestus* (7.89 vs 8.16%; χ^2^ = 2.58^e−29^; P = 1). The majority of both infected vectors with malaria parasites harboured *Plasmodium falciparum* (93.65% for *An. gambiae* and 98. 31% for *An. funestus*). Overall, the EIR range for both species in the different districts appeared to be high (0.35–2.20 infected bites per human per night) with the highest value observed in the district of North-Eastern-Bouaké. There were no significant differences between transmission occurring outdoor and indoor for both species. Of the *An. gambiae s.l*. analysed, only *An. gambiae *sensu stricto (14.1%) and *Anopheles coluzzii* (85.9%) were found. The allelic frequencies of *kdr* and *ace-1*^*R*^ were higher in *An. gambiae* (0.97 for *kdr* and 0.19 for *ace-1*^*R*^) than in *An. coluzzii* (0.86 for *kdr* and 0.10 for *ace-1*^*R*^) (P < 0.001).

**Conclusion:**

Despite universal coverage with long-lasting insecticidal nets (LLINs) in the area, there was an abundance of the malaria vectors (*An. gambiae* and *An. funestus*) in the study area in central Côte d’Ivoire. Consistent with high insecticide resistance intensity previously detected in these districts, the current study detected high *kdr* frequency (> 85%), coupled with high malaria transmission pattern, which could guide the use of Eave tubes in the study areas.

## Background

Malaria is caused by protozoan parasites belonging to the *Plasmodium* genus, which are transmitted by the female *Anopheles* mosquito during blood feeding. Over the last 10 years, considerable efforts have been made to control malaria in many parts of the world, especially in sub-Saharan Africa. This has led to the decline in malaria transmission in many parts of Africa [1, 2]. According to the last World Malaria Report [3], the significant progress in malaria control can be attributed to a scale-up of vector control interventions, as well as improved diagnostic testing, rapid and efficient treatment of malaria patients. However, despite these considerable efforts to reduce transmission, malaria remains one of the major causes of morbidity and mortality in sub-Saharan Africa [1, 4]. Vector control relies on a handful of insecticides used for indoor residual spraying (IRS) and treatment of long-lasting insecticidal nets (LLINs) and insecticide resistance has been widely detected in malaria vectors across the continent [5–8]. The situation is particularly worrying with an increase in intensity and mechanisms of insecticide resistance detected over time [8, 9]. There is a pressing need for effective, sustainable tools or strategies for malaria control.

The observation that host-seeking African malaria vectors predominantly enter human dwellings through open eaves motivated the development of the EaveTubes technology [10]. EaveTubes are an innovative delivery system where insecticide-treated inserts are placed in tubes installed in the eaves of houses. These inserts enable the transfer of a high dose of insecticide capable of killing even strongly insecticide-resistant *Anopheles* mosquitoes [11]. EaveTubes, in combination with screening of windows and doors, were found to reduce malaria transmission in a cluster-randomized controlled trial (CRT) conducted in central Côte d’Ivoire between 2016 and 2019 [12]. EaveTubes present a mechanism to expose the mosquito population to alternative classes of insecticide presenting a delivery method that could be utilized for insecticide resistance management [10, 11].

Collecting baseline data on entomological parameters, including vector densities, malaria sporozoite rates and insecticide resistance phenotypes, would be valuable data that will justify the choice for EaveTubes as appropriate intervention in the area. The current study was conducted prior to the start of the CRT across all study villages in central Côte d’Ivoire.

## Methods

### Study site

The study was conducted in 40 villages across five health districts (Béoumi, Southern-Bouaké, North-Eastern-Bouaké, North-Western-Bouaké, Sakassou). All districts were covered with a high rate (> 80%) of standard pyrethroids-based LLINs (Permanet 2.0 and OlysetNet). Malaria transmission in these areas occurs year-round with a peak during the wet season (April-November). The main malaria vector, *Anopheles gambiae *sensu lato (*s.l*.) was highly resistant to almost all public health classes of insecticides [13], with 125.8 bites per human per night and entomological inoculation rates (EIR), reaching 459.9 infected bites per human per night in some rural places of the districts [14]. *Anopheles funestus s.l.* and *Anopheles nili s.l.* were also present, but less abundant.

For the CRT, 40 village (clusters) were identified within a 60 km radius around the city of Bouaké. The villages were selected to have 100–600 houses, of which at least 80% had corrugated iron roof and brick-made walls, suitable for installation of EaveTubes. Villages were at least 2 km apart from each other.

### Mosquito collection

To assess malaria transmission indicators, a cross-sectional survey was conducted between November and December 2016 (the beginning of the dry season), to collect adult mosquitoes within homes by human landing catches (HLC). Volunteers were recruited within the study villages. They sat with their legs uncovered attracting mosquitoes around and collecting those landing on their legs using glass haemolysis tubes plugged with cotton. Captures were done in each village over two consecutive nights by two mosquito collectors (one indoors and one outdoors) in five randomly selected households. For each capture point, one volunteer collected mosquitoes from 18:00 to 00:00 h and a second volunteer took over from 00:00 to 08:00. Volunteers rotated from a capture point to another to account for any possible differences in individual attractiveness to mosquitoes. The mosquitoes collected were kept in cool boxes and transported to the laboratory for processing the next morning.

### Identification and processing of mosquitoes

Mosquitoes were first identified using morphological identification key [15]. Only known malaria vector species in Côte d’Ivoire (*An. gambiae* and *An. funestus*) [14] were analysed, although other rare *Anopheles* with potential for malaria transmission were collected. Due to the large numbers of *An. gambiae* and *An. funestus* captured during the HLC, only a sub-set of samples was analysed.

For this sub-set, two to four females of *An. gambiae* and *An. funestus* were randomly selected per sampling hour and per site and their ovaries were dissected to determine parity status [16]. Of the parous female mosquitoes, up to 60 per village, when available, were randomly selected to be processed for sporozoite infection by quantitative polymerase chain reaction (qPCR) assay [17]. The same sub-sample was also tested for molecular identification of species [18] and to detect the Knockdown resistance gene L1014F (*kdr-w*) [19] and the acetylcholinesterase gene G119S (*ace-1*^*R*^) mutations [20].

### Data analysis

Indoor and outdoor human biting rates (HBR) measured were the mean number of vector bites received per person per night of collection (b/p/n). The result was obtained by the number of anophelines captured at each sampling point divided by the total number of sampling nights and the average number of collectors. Parity rate (PR) was the proportion of parous mosquitoes over the total dissected. The *Plasmodium sporozoite* rate (SR) in each vector species population was the number of mosquitoes infected with sporozoites in the head-thorax, divided by the total number of mosquitoes tested. The nightly EIR was the number of infectious bites per person per night and defined as the product of HBR and SR. It is conventionally the product of the daily HBR and the SR from the caught mosquitoes. For this study, nightly EIR was calculated using the following formula:

1 $$EIR=HBR*SR$$

2 $$E{\text{IR}} = \left[ {\left( {\frac{{{\text{Total\;vector\;caught}}}}{{{\text{Total\;capture\;night}}}}} \right)*\left( {\frac{{{\text{Total\;sporozoite\;positive}}}}{{({\text{Parous\;tested}} + {\text{non}} - {\text{parous}})}}} \right)} \right]$$

3 $$\mathrm{Parous \; tested}+\mathrm{non}-\mathrm{parous}=\frac{\mathrm{Parous}}{\mathrm{Parity rate}}$$

In (2), the first (x) term is $HBR$ and the second (y) is $SR$. This approach was used because the SR was estimated assuming that all non-parous mosquitoes were sporozoite negative.

Data were analysed in R (version 4.0.3). The Wilcoxon (W) test was used to compare the differences in vector species for HBR and EIR between sampling locations in households and among health districts. The Pearson’s Chi-square (χ^2^) test was used to compare parity and sporozoite rates. For all statistics, a p value below 0.05 was considered as statistically significant.

The allelic frequencies of the two resistance genes (*kdr* L1014F and *ace-1*^*R*^ G119S) in *An. gambiae* sibling species were tested to Hardy–Weinberg equilibrium (HWE) conformity using the exact HW test and also compared.

### Ethics clearance

This study followed the ethics principles recommended by the Côte d’Ivoire Ministry of Health ethics committee (ref: 039/MSLS/CNER-dkn), the Pennsylvania State University’s Human Research Protection Program under the Office for Research Protections (ref.: STUDY00003899 and STUDY00004815), and the London School of Hygiene and Tropical Medicine ethical review board (No. 11223).

Verbal and written informed consent from all participants were obtained in the local language prior to their enrolment in the study. Volunteer mosquito collectors were well trained on how to collect mosquitoes without being bitten. They received vaccination against yellow fever and the project offered treatment of confirmed malaria cases free of charge, according to the national malaria control programme policy.

## Results

### Mosquito species composition, density and human biting pattern

**Fig. 1:**
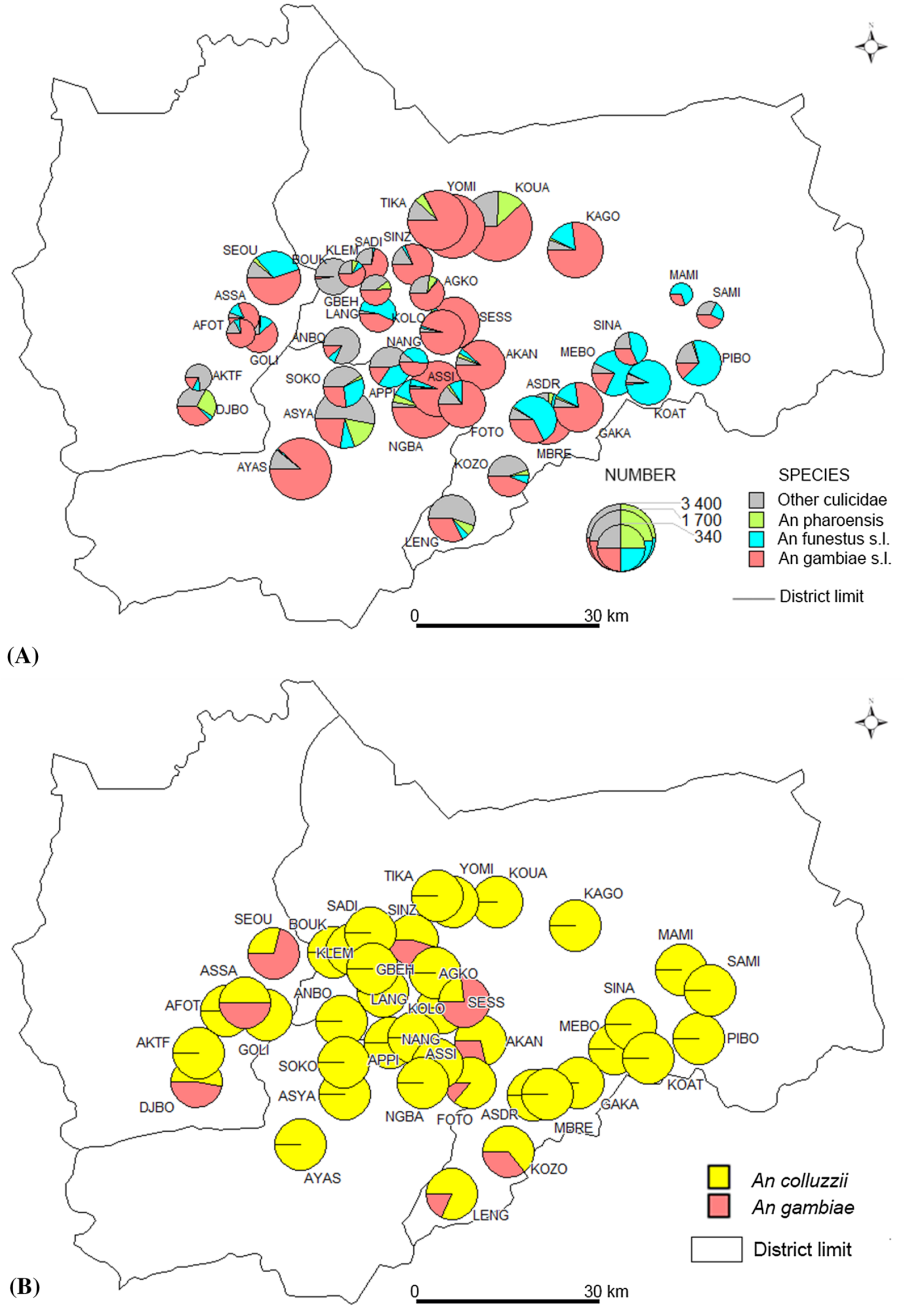
Map of mosquito densities and composition in the 40 village-clusters of the study area. **A** Overall mosquito density; **B** *An. gambiae* s.l. species complex distribution in the forty (40) villages

**Fig. 2:**
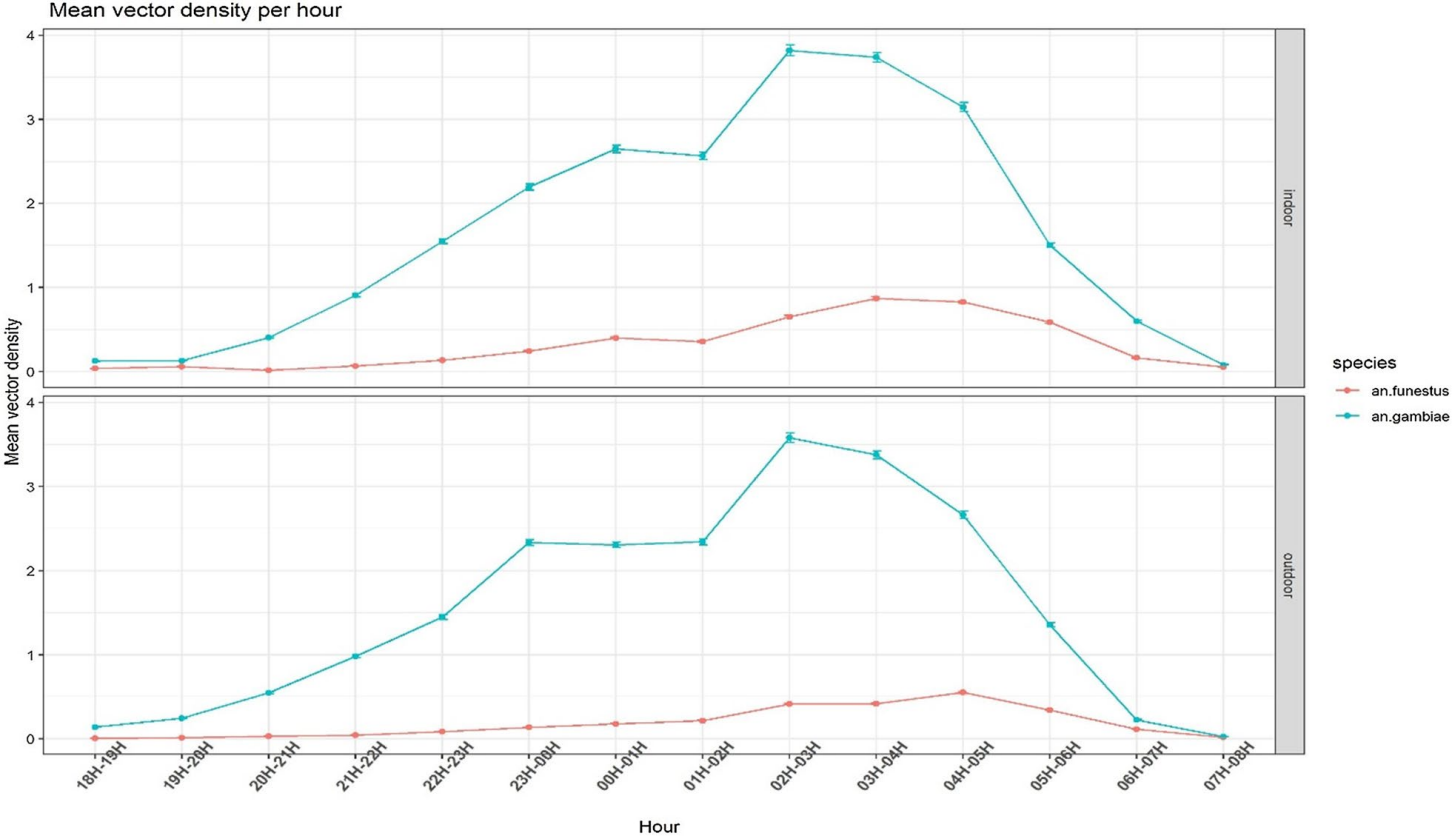
Hourly outdoor and indoor biting profiles of *An. gambiae* s.l. and *An. funestus* s.l. across all the study villages. Points show mean and bars indicate hourly change in number of mosquito bites

A total of 15,632 female mosquitoes were captured using HLC, of which 66.2% (10,350) were *An. gambiae* and 1,615 (10.3%) were *An. funestus* (Table 1 and Fig. 1A). There was a relatively equal preference towards biting both indoors and outdoors for both vectors and began biting from the early evening (from 19.00 onwards) to reach a peak around 02.00 (*An. gambiae)* or 03:00 (*An. funestus*) (Fig. 2). Biting then decreased steadily, and by dawn (06:00) it fell below 0.2 b/p/n. Overall, the mean biting rate for *An. gambiae* (22.13 b/p/n) was significantly higher (six-fold) than that for *An. funestus* (3.51 b/p/n) (P < 0.01) and this was evident both indoors and outdoors, except in North-eastern-Bouaké (8.4 vs 6.34 b/p/n; W = 2,236.5, P = 0.368) (Table 2). Overall, the biting patterns indoors and outdoors were similar for *An. gambiae* and *An. funestus* (P > 0.05) (Table 2).

**Table 1:** Number of mosquitoes collected by human landing catch (HLC) between November–December 2016

Mosquito species	Number of females collected (%)	Collection location	
		Number indoor (%)	Number outdoor (%)
*An*. *gambiae* s.l	10350 (66.2)	5714 (55.2)	4636 (44.8)
*An. funestus* s.l	1615 (10.3)	1034 (64.0)	581 (36.0)
Other *Anopheles spp*	894 (5.7)	428 (47.9)	466 (52.1)
*Mansonia sp.*	1990 (12.7)	1,074 (54.0)	916 (46.0)
*Culex sp.*	764 (4.9)	380 (49.7)	384 (50.3)
*Aedes sp.*	19 (0.1)	13 (68.4)	6 (31.6)
Total	15632 (100)	8643 (55.3)	6989 (44.7)

**Table 2:** Variation of human biting rate (HBR) in five districts in Gbêkê region, central Côte d’Ivoire between November–December 2016

District	Indoor		Outdoor		Indoor + Outdoor	
	N_1_	HBR (b/h/n) [95% CI]	N_1_	HBR (b/h/n) [95% CI]	N_1_	HBR (b/h/n) [95% CI]
Béoumi						
*An. gambiae* s.l	184	5.26 [2.78–7.73]	248	7.09 [4.13–10.04]	432	6.17 [4.28–8.06]
*An. funestus* s.l	85	2.43 [0.87–3.99]	108	3.09 [1.32–4.85]	193	2.76 [1.61–3.91]
Pooled values	269	7.69 [3.96–11.41]	356	10.17 [5.62–14.72]	625	8.93 [6.05–11.81]
Southern-Bouaké						
*An. gambiae* s.l	415	13.83 [7.86–19.81]	295	9.83 [6.18–13.48]	710	11.83 [8.40–15.27]
*An. funestus* s.l	290	9.67 [4.33–15.01]	160	5.33 [2.92–7.74]	450	7.50 [6.60–10.40]
Pooled values	705	23.50 [15.47–31.53]	455	15.17 [10.35–19.99]	1160	19.33 [14.66–24.00]
North-Eastern-Bouaké						
*An. gambiae* s.l	251	7.17 [3.52–10.82]	338	9.66 [4.12–15.19]	589	8.41 [5.17–11.66]
*An. funestus* s.l	317	9.06 [4.62–13.49]	127	3.63 [2.26–5.00]	444	6.34 [3.99–8.70]
Pooled values	568	16.23 [10.62–21.83]	465	13.28 [7.49–19.08]	1033	14.76 [10.81–18.70]
North-Western-Bouaké						
*An. gambiae* s.l	2770	34.62 [22.97–46.27]	2485	31.06 [20.72–41.40]	5255	32.84 [25.13–45.55]
*An. funestus* s.l	44	0.55 [0.20–0.90]	45	0.56 [0.24–0.88]	89	0.56 [0.32–0.79]
Pooled values	2814	35.17 [23.51–46.84]	2530	31.62 [21.30–41.95]	5344	34.40 [25.69–41.11]
Sakassou						
*An. gambiae* s.l	1766	35.32 [23.69–46.95]	1598	31.96 [22.49–41.43]	3364	33.64 [26.26–41.01]
*An. funestus* s.l	289	5.78 [3.17–8.39]	150	3.00 [1.93–4.07]	439	4.39 [2.98–5.80]
Pooled values	2055	41.10 [28.65–53.55]	1748	34.96 [25.38–44.54]	3803	38.03 [30.29–45.77]
Overall						
*An. gambiae* s.l	5386	23.42 [18.32–28.51]	4964	21.58 [17.14–26.03]	10,350	22.50 [19.13–25.87]
*An. funestus* s.l	1025	4.46 [3.25–5.66]	590	2.56 [2.01–3.12]	1615	3.51 [2.85–4.18]
Pooled values	6411	27.87 [22.65–33.10]	5554	24.15 [19.69–28.61]	11,965	26.01 [22.28–29.44]

*N*_*1*_ number of specimens collected, *HBR* human biting rate. *b/h/n* Bites per human per night. For the HBR distribution, values between *An. gambiae* s.l. and *An. funestus* s.l. were significantly different overall (Wilcoxon test; P < 0.05). There were no significant differences between outdoor and indoor HBR for both species (Wilcoxon test; P > 0.05). *CI* confidence interval

### Parity rate

Parity rates were high for both species caught indoors and outdoors; it averaged 89–91% for *An. gambiae* and 97–98% for *An. funestus*, with overall a significant difference (P > 0.05) between the two species. There were no significant differences in the parity rates indoors and outdoors across health districts (P > 0.05) (Table 3).

**Table 3:** Variation of parity rate (PR) in five districts in Gbêkê region, central Côte d’Ivoire between November–December 2016

District	Indoor		Outdoor		Indoor + Outdoor	
	N_2_	PR (%) [95% CI]	N_2_	PR (%) [95% CI]	N_2_	PR (%) [95% CI]
Béoumi						
*An. gambiae* s.l	130	93.84 [87.84–97.11]	160	91.87 [86.22–95.42]	290	92.76 [88.98–95.35]
*An. funestus* s.l	12	100.00 [69.87–100]	19	94.74 [71.89–99.72]	31	96.77 [81.49–99.83]
Pooled values	142	94.37 [88.83–97.36]	179	92.18 [86.87–95.50]	321	93.15 [89.66–95.56]
Southern-Bouaké						
*An. gambiae* s.l	289	94.46 [90.98–96.70]	210	94.76 [90.57–97.22]	499	94.59 [92.12–96.34]
*An. funestus* s.l	241	98.75 [96.10–99.68]	136	98.53 [94.25–99.74]	377	98.67 [96.75–99.51]
Pooled values	530	96.41 [94.36–97.77]	346	96.24 [93.50–97.90]	876	96.34 [94.82–97.44]
North-Eastern-Bouaké						
*An. gambiae* s.l	111	90.99 [83.66–95.36]	133	85.71 [78.34–90.96]	244	88.11 [83.22–91.77]
*An. funestus* s.l	261	99.23 [96.95–99.87]	101	97.03 [90.93–99.23]	362	98.62 [96.62–99.49]
Pooled values	372	96.77 [94.28–98.24]	234	90.60 [85.92–93.88]	606	94.39 [92.17–96.03]
North-Western-Bouaké						
*An. gambiae* s.l	801	88.51 [86.05–90.60]	797	90.34 [88.02–92.26]	1598	89.42 [87.78–90.87]
*An. funestus* s.l	41	100.00 [89.33–100]	35	97.14 [83.38–99.85]	76	98.68 [90.89–99.93]
Pooled values	842	89.07 [86.72–91.06]	832	90.62 [88.39–92.47]	1674	89.84 [88.27–91.23]
Sakassou						
*An. gambiae* s.l	683	92.24 [89.91–94.08]	670	86.86 [84.02–89.28]	1353	89.58 [87.79–91.13]
*An. funestus* s.l	242	95.85 [92.27–97.88]	102	99.02 [93.88–99.98]	344	96.80 [94.18–98.31]
Pooled values	925	93.19 [91.32–94.69]	772	88.47 [85.95–90.59]	1697	91.04 [89.56–92.34]
Overall						
*An. gambiae* s.l	2014	91.11 [89.76–92.30]	1970	89.44 [87.98–90.74]	3984	90.29 [89.31–91.18]
*An. funestus* s.l	797	98.12 [96.84–98.90]	393	97.96 [95.87–99.05]	1190	98.07 [97.07–98.74]
Pooled values	2811	93.10 [92.08–93.99]	2363	90.86 [89.61–91.97]	5174	92.07 [91.30–92.79]

*N*_*2*_ number of *Anopheles* specimens dissected, *PR* parity rate. For the PR distribution, values between *An. gambiae* s.l. and *An. funestus* s.l.  were significantly different overall (Chi-square test; P < 0.05). There were no significant differences between outdoor and indoor PR for both species (Chi-square test; P > 0.05). *CI* confidence interval

### Plasmodium sporozoite rate

Overall, infection rate for *An. funestus* (8.01%) was significantly higher (two-fold) than for *An. gambiae* (4.14%) (χ^2^ = 12.216; P < 0.0001). There was no significant difference between the proportion infected outdoors and indoors for *An. gambiae* (4.03 vs 4.13%; χ^2^ = 0.011; P = 0.9197), and for *An. funestus* (7.89 vs 8.16%; χ^2^ = 2.58^e−29^; P = 1) (Table 4).

**Table 4:** Variation of sporozoite rate (SR) in five districts in Gbêkê region, central Côte d’Ivoire between November–December 2016

District	Indoor			Outdoor			Indoor + Outdoor		
	N_3_	n	SR (%) [95% CI]	N_3_	n	SR (%) [95% CI]	N_3_	n	SR (%) [95% CI]
Béoumi									
*An. gambiae* s.l	87.38	4	4.57 [1.48–11.95]	87.08	2	2.30 [0.40–8.83]	174.64	6	3.43 [1.40–7.67]
*An. funestus* s.l	10	0	0.00 [0.00–0.00]	18.99	2	10.53 [1.84–34.54]	28.93	2	6.91 [1.21–24.26]
Pooled values	97.49	4	4.10 [1.32–10.77]	106.31	4	3.76 [1.21–9.91]	203.97	8	3.92 [1.83–7.86]
Southern-Bouaké									
*An. gambiae* s.l	225.49	8	3.55 [1.65–7.13]	91.81	4	4.36 [1.40–11.40]	317.16	12	3.78 [2.06–6.69]
*An. funestus* s.l	120.51	8	6.64 [3.12–13.07]	103.52	5	4.83 [1.79–11.44]	223.98	13	5.81 [3.26–9.95]
Pooled values	344.36	16	4.65 [2.77–7.59]	196.38	9	4.58 [2.25–8.80]	540.79	25	4.62 [3.07–6.84]
North-Eastern-Bouaké									
*An. gambiae* s.l	62.64	9	14.37 [7.18–26.03]	56.00	4	7.14 [2.31–18.12]	119.17	13	10.91 [6.17–18.26]
*An. funestus* s.l	106.82	20	18.72 [12.07–27.67]	81.42	13	15.97 [9.10–26.12]	187.59	33	17.59 [12.5–23.97]
Pooled values	168.44	29	17.22 [12.01–23.96]	140.18	17	12.13 [7.43–18.97]	307.23	46	14.97 [11.27–19.57]
North-Western-Bouaké									
*An. gambiae* s.l	274.54	6	2.18 [0.89–4.93]	274.52	16	5.83 [3.48–9.47]	549.09	22	4.01 [2.59–6.10]
*An. funestus* s.l	36	2	5.55 [0.97–20.01]	32.94	1	3.03 [0.16–17.54]	68.91	3	4.35 [1.13–13.03]
Pooled values	313.24	8	2.55 [1.19–5.16]	308.98	17	5.50 [3.34–8.83]	622.22	25	4.02 [2.67–5.96]
Sakassou									
*An. gambiae* s.l	187.55	7	3.73 [1.64–7.85]	164.63	3	1.82 [0.47–5.65]	352.76	10	2.83 [1.45–5.32]
*An. funestus* s.l	132.5	2	1.51 [0.26–5.90]	93.92	6	6.39 [2.62–13.92]	227.27	8	3.52 [1.64–7.07]
Pooled values	321.92	9	2.79 [1.37–5.42]	266.76	9	3.37 [1.65–6.52]	588.75	18	3.06 [1.88–4.88]
Overall									
*An. gambiae* s.l	842.94	34	4.03 [2.85–5.65]	677.55	29	4.28 [2.93–6.16]	1521.76	63	4.14 [3.22–5.30]
*An. funestus* s.l	405.62	32	7.89 [5.54–11.06]	330.75	27	8.16 [5.54–11.79]	736.21	59	8.01 [6.20–10.27]
Pooled values	1252.42	66	5.27 [4.13–6.69]	1023.55	56	5.47 [4.19–7.09]	2276.53	122	5.36 [4.49–6.38]

*N*_*3*_ number of *Anopheles* parous (P) examined plus relevant non-parous specimens (NP), *SR* sporozoite rate, *n* number of *Anopheles* species infected. For the SR distribution, values between *An. gambiae* s.l. and *An. funestus* s.l. were significantly different overall (Chi-square test; P < 0.05). There were no significant differences between outdoor and indoor SR for both species (Chi-square test; P > 0.05). *CI* confidence interval

**Fig. 3:**
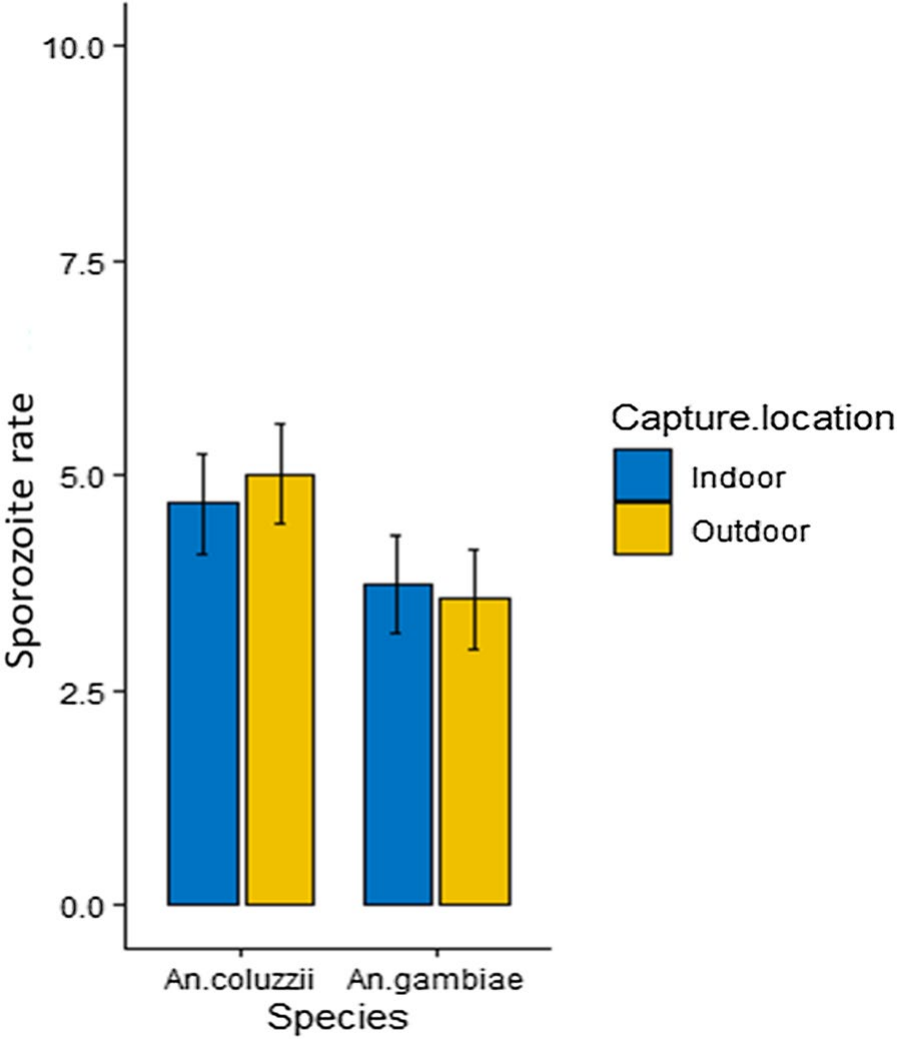
Sporozoite rate in *Anopheles gambiae* complex. Error bars represent 95% confidence intervals (CIs)

The majority of *An. gambiae* infected with malaria parasites harboured *Plasmodium falciparum* (93.65%), and a few had *Plasmodium malariae* (6.35%) (Table 5). There was no *Plasmodium ovale* detected in any of the samples tested for *An. gambiae*. Almost all *An. funestus* analysed were infected with *P. falciparum* (98.31%) and only one individual had *P. ovale* (1.69%), with no *An. funestus* testing positive for *P. malariae* (Table 5). Within the *An. gambiae* complex, the proportions of sporozoite rate in parous individuals for *An. gambiae *sensu stricto (*s.s.*) were similar to *Anopheles coluzzii* (P > 0.05) (Fig. 3).

**Table 5:** Sporozoite infection rate (SR) and malaria parasites

Species	N_1_	N_3_	n	% SR [95% CI]	*Plasmodium* species per vector [95% CI]					
					*P. falciparum*		*P. malariae*		*P. ovale*	
					p	% [95% CI]	p	% [95% CI]	p	% [95% CI]
*An. gambiae* s.l	10,350	1521.76	63	4.14 [3.22–5.30]	59	93.65 [71.29–120.8]	4	6.35 [1.73–16.26]	0	0.00 [0.00–0.00]
*An. funestus* s.l	1615	736.21	59	8.01 [6.20–10.27]	58	98.31 [74,65–127.08]	0	0.00 [0.00–0.00]	1	1.69 [0.04–9.44]
Overall	11,965	2276.53	122	5.36 [4.49–6.38]	117	95.90 [79.31–114.94]	4	3.28 [0.93–8.39]	1	0.82 [0.02–0.4 l]

For abbreviations of *N*_*1*_, *N*_*3*_ and *n*, see Tables 2 and 4, *p* total number of positive specimens to *Plasmodium* spp

### Entomological inoculation rate

The EIRs ranged 0.21–2.20 for *An. gambiae* and 0.02–1.11 for *An. funestus* across health districts. The overall transmission for *An. gambiae* (0.77 ib/h/n) was two-fold higher than for *An. funestus* (0.38 ib/p/n) (W = 1,263; P = 3.92.10^–06^), without differences indoors and outdoors with either species (P > 0.05) (Table 6).

**Table 6:** Variation of entomological inoculation rate (EIR) in five districts in Gbêkê region, central Côte d’Ivoire between November–December 2016

District	Indoor	Outdoor	Indoor + Outdoor
	EIR (ib/h/n)	EIR (ib/h/n)	EIR (ib/h/n)
Béoumi			
*An. gambiae* s.l	0.24	0.16	0.21
*An. funestus* s.l	0	0.32	0.19
Pooled values	0.31	0.38	0.35
Southern-Bouaké			
*An. gambiae* s.l	0.49	0.43	0.45
*An. funestus* s.l	0.64	0.25	0.43
Pooled values	1.09	0.69	0.89
North-Eastern-Bouaké			
*An. gambiae* s.l	1.03	0.69	0.92
*An. funestus* s.l	1.69	0.58	1.11
Pooled values	2.79	1.61	2.2
North-Western-Bouaké			
*An. gambiae* s.l	0.76	1.81	1.31
*An. funestus* s.l	0.03	0.02	0.02
Pooled values	0.89	1.74	1.38
Sakassou			
*An. gambiae* s.l	1.32	0.58	0.95
*An. funestus* s.l	0.09	0.19	0.15
Pooled values	1.15	1.18	1.16
Overall			
*An. gambiae* s.l	0.78	0.73	0.77
*An. funestus* s.l	0.49	0.27	0.38
Pooled values	1.25	1.12	1.2

*EIR* entomological inoculation rate. i*b/h/n* infected bites per human per night. For the EIR distribution, values between *An. gambiae* s.l.and *An. funestus* s.l. were significantly different overall (Wilcoxon test P < 0.05). There were no significant differences between outdoor and indoor EIR for both species (Wilcoxon test; P > 0.05). *CI* confidence interval

### Frequencies of the *kdr 1014F* and *ace-1*.^*R*^* 119S *alleles in *Anopheles gambiae* species complex

Out of 1,374 *An. gambiae* s.l. mosquitoes analysed by PCR, 1,350 were successfully identified to species (< 2% failure rate). Both *An. gambiae s.s.* (n = 190; 14.1%) and *An. coluzzii* (n = 1,160; 85.9%) were found within the *An. gambiae* complex analysed. For both *kdr* and *ace-1*^*R*^ genes, the allelic frequencies were higher in parous individuals of *An. gambiae s.s.* than in *An. coluzzii* (P < 0.001) (Table 7).

**Table 7:** *Kdr L1014F* and *ace-1*^*R*^* G119S* mutation frequencies in *Anopheles gambiae* s.l. populations

Mutation	Species	N	SS (%)	RS (%)	RR (%)	Allelic frequency	p(HW)
*Kdr L1014F*	*An. coluzzii*	1 145	43 (3.75)	233 (20.35)	869 (75.89)	0.861^a^	0.000
	*An. gambiae*	187	1 (0.53)	9 (4.81)	177 (94.65)	0.971^b^	0.140
*Ace-1 G119S*	*An. coluzzii*	1 142	949 (83.10)	148 (12.96)	45 (3.94)	0.104^a^	0.000
	*An. gambiae*	185	121 (65.40)	55 (29.73)	9 (4.86)	0.197^b^	0.362

*N* number of mosquitoes genotyped; SS: susceptible; *RS* heterozygote; *RR* resistant; *p (HW)* exact Hardy–Weinberg test P-value. for each mutation, allelic frequencies with different superscript letters (*a* and *b*) differ significantly between species (G-test, P < 0.05)

## Discussion

Here we have provided a descriptive analysis of the entomological indicators relevant to malaria transmission in central Côte d’Ivoire, prior to the start of a CRT evaluating a new malaria vector control intervention.

The human malaria vector species that were found in the study area at the time of sampling (November–December 2016) were *An. gambiae* and *An. funestus*, with *An. gambiae* being more abundant. The predominance of *An. gambiae* could be explained by the presence of breeding sites favourable to *An. gambiae* (e.g., rice paddy fields, vegetable plots, marshes) throughout the study area [21–23].This aligns with previous studies conducted in the same area, and elsewhere in Côte d’Ivoire, which reported the predominance of *An. gambiae* among local malaria vectors [24, 25]. With *An. funestus*, swampy marshes along rivers were the main breeding sites as also observed in previous study in the areas [26].

*Anopheles gambiae s.s.* and *An. coluzzii* were the only members of *An. gambiae* complex identified in the study area. *Anopheles coluzzii* found in high proportion (85.90%) was consistent with previous findings in the area of Bouaké [13, 23, 27] but contrasts with other studies in the northern savannah of the country, where *An. gambiae* was more prevalent [24, 28]. The difference observed is likely due to variations in mosquito larval habitats; *An*. *coluzzii* tends to exploit more permanent breeding sites, including those created by the type of irrigation for rice cultivation found in Bouaké and the surrounding area. Permanent availability of breeding sites, due to intensive and perennial agricultural practices could have led to the presence of *An. coluzzii* [29].

The increase in biting activity for both species coinciding with the time when many people would be going to bed was found with a peak in biting around 02:00 for *An. gambiae* and 03:00 for *An. funestus*. This is similar to previous entomological studies conducted in same area around Bouaké [22] as well as the northern part of Cote d’Ivoire [24, 30] and elsewhere in Africa [31]–33]. These biting profiles highlight the utility of LLINs as a personal protective measure against host-seeking malaria vectors. However, the fact that outdoor biting *An. gambiae* mosquitoes were found in similar proportion to indoor biting mosquitoes is a sign that people are at risk of malaria transmission when they are outside in the evenings. It further highlights the need for novel strategies or tools to target outdoor malaria transmission [34, 35].

Mean parity rates and sporozoite rates were high in both species, especially in *An. funestus*, indicating a high prevalence of older female mosquitoes, which had already gone through several cycles of blood feeding. Despite lower numbers, the overall sporozoite index rate for *An. funestus* was higher than *An. gambiae*, indicating that it is still an important malaria vector in the area. These results are consistent with findings from previous studies in northern Côte d’Ivoire [24, 30], and show a need to better characterize the biology and ecology of *An. funestus* in this area [26], as well as careful monitoring of the epidemiological significance of *An. funestus* in malaria transmission.

The mean nightly EIR for both species in this study was 1.20 infected bites per person per night between November and December 2016. By extrapolation, this global nightly estimated infected bites could correspond to 438 infected bites per person per year. Meta-analysis from a pool of studies conducted in various epidemiological settings across Africa reported EIRs ranging 1 to 1,000 infected bites per person per year and that an annual EIR high than 200 per person per year was consistently associated with malaria prevalence averaging > 80% [31]. Similarity, in a baseline epidemiological study conducted at a similar time, in the same area, prevalence was reported to be 73.9% [12]. The area around Bouaké can therefore be considered as highly endemic for malaria. Moreover, EIR in the study area was equally high indoors and outdoors and varied across health districts in both vector species, possibly linked to the high vector abundance in the area [14]. The similarity between indoor and outdoor transmission of malaria is inconsistent with LLIN use in the area [9].

Consistent with recent studies carried out in the area of Bouaké [7, 9, 13, 36], there was a high frequency of both *kdr* and *ace1*^*R*^ genes in *An. gambiae* and *An. coluzzii,* with a higher frequency for *An. gambiae*; probably due to selection pressure through the use of insecticide. The lower frequency the resistance alleles in *An. coluzzii* was associated with higher proportion of heterozygous, implying that *An. gambiae* is better adapted to insecticide pressure as evidenced elsewhere in Côte d’Ivoire [8, 37] and other parts of sub-Saharan Africa [24, 38, 39].

Resolving the problem posed by outdoor transmission of malaria has become critical [34, 40] LLINs and IRS are effective strategies controlling malaria but unfortunately they can only operate indoors [41, 42]. Once again the high outdoor transmission of malaria in this study triggers the urgent search for innovative tools or strategies to overcome outdoor transmission of malaria.

## Conclusion

Densities of *An. gambiae* and *An. funestus* were high in central Côte d’Ivoire prior to the start of a CRT evaluating a new method of malaria vector control. The density of *An. gambiae* was higher than for *An. funestus*, although *An. funestus* had overall higher rate of infection with *Plasmodium* parasites (sporozoite index). However, malaria transmission indicator based on the number of infected bite per person per night (EIR) for *An. gambiae* was consistently higher than for *An. funestus*, without differences indoors and outdoors with either species, despite universal coverage of LLINs in the area. Owing to its resistance breaking potential, the claim is to evaluate EaveTubes in areas of high insecticide resistance and where the force of malaria transmission is intense.

Consistent with high insecticide resistance intensity previously detected in these districts, the current study detected high Kdr frequency (> 85%), coupled with high malaria transmission pattern, which could guide the use of EaveTubes in the study areas.

## Data Availability

The datasets supporting the conclusions of this manuscript are included within the manuscript and its additional files, and are available from the corresponding author on reasonable request.

## Abbreviations

*Ace-1*^*R*^: Acetylcholinesterase-1 resistance
*ace-1*^*R*^ G119S: G119S mutation in a*ce-1*^*R*^
CRT: Cluster-Randomized controlled Trial
EIR: Entomological Inoculation Rate
HBR: Human Biting Rates
HLC: Human Landing Catches
HWE: Hardy–Weinberg Equilibrium
IPR: Institut Pierre Richet
IRS: Indoor Residual Spraying. L1014F *kdr*: West knockdown resistance
LLINs: Long Lasting Insecticidal Nets
OR: Odds ratio
PR: Parity Rate
qPCR: Quantitative Polymerase Chain Reaction
R: Resistant
S: Susceptible
SR: Sporozoite Rate
VCPEC: Vector Control Evaluation Centre
WHO: World Health Organization
